# 

**Published:** 1874-12

**Authors:** 


### Contents of December Number.

### ORIGINAL DEPARTMENT.

ART. I.—Abstract of Proceedings of the Buffalo Medical Association. Reported by W. W. Miner, M. D., Secretary, pro tem............. 161
ART. II.—Albany County Medical Society, Semi-Monthly Meeting. Reported by F. C. Curtis, M. D.,.................................. 170
ART. III.—Semi-Monthly Meeting Rochester Medical Society. Reported by J. O. Roe, M. D., Secretary,............................. 174
ART. IV.—Clinical Remarks in the Buffalo Hospital of the Sisters of Charity, by Prof. J. F. Miner, M. D. Reported by W. W. Miner, M. D.................................................... 181
ART. V.—Report of Surgical cases at the Buffalo General Hospital, with Clinical Remarks, by Prof. J. F. Miner, M. D. Reported by Bernard Bartow, M. D., and W. W. Miner, M. D.,............. 186

### MISCELLANEOUS.

Public Health,.............................................. 190
Death following Vaginal Injection.................................. 193
Vivisection,................................................ 193

### EDITORIAL DEPARTMENT.

The Alumni Association of the Medical Department of the University of Buffalo,............................................ 194
To Our Subscribers,........................................ 195
The N. Y. Observer,........................................ 195
Lloyd's Maps,.............................................. 195
Meeting of the Alumni of the Albany Medical College, ............ 195
Erie County Medical Society,.................................. 196

BOOKS REVIEWED :

A Practical Treatise on Diseases of Women. By T. Gailiard Thomas, M. D.......................................... 196
Therapeutics and Materia Medica. By Alfred Stillé, M. D.,.. 197
Clinical Lectures. By N. S. Davis, M. D.,.................. 198
A Guide to the Practical Examination of Urine. By James Tyson, M. D.,........................................ 198
Lindsay & Blakiston's Visiting List,........................ 199
Virginia Medical Monthly, ................................. 199
The Breath, and Diseases which give it a Fetid Odor. By J. W. Howe, M. D.,...................................... 199
Clinical Lectures on Diseases of the Nervous System. By Wm. H. Hammond, M. D.,................................ 200

Books and Pamphlets Received ................................. 200

---

THE BUFFALO

